# The Conundrum of Septic Shock Imitators in Patients with Hematologic Cancers: Case Presentation and Possible Differential Diagnoses

**DOI:** 10.1155/2019/6561018

**Published:** 2019-09-10

**Authors:** Giorgio Berlot, Giulia Moratelli, Martina Tarchini, Katiuscia Battaglia, Paolo Grassi, Nadia Zarrillo, Vincenzo Colella, Rossana Bussani

**Affiliations:** ^1^Department of Anesthesia and Intensive Care, University of Trieste, Italy; ^2^Department of Anesthesia and Intensive Care, Azienda Ospedaliera Sant'Anna e San Sebastiano di Caserta, Italy; ^3^Department of Pathology, University of Trieste, Italy

## Abstract

The authors describe the case of a patient treated with several cycles of chemotherapy due to an advanced stage non-Hodgkin lymphoma. One daafter the last cycle, he was admitted to our Intensive Care Unit with a septic shock-like clinical picture which didn't respond to the aggressive treatment and the patient died a few hours later. The autoptical findings cast some doubts on the diagnosis, and demonstrated the presence of other factors imitating its symptoms. In this article, the mimickers of septic shock are reviewed and discussed, as some of them require an aggressive immunosuppression instead of the recommended treatment for septic shock.

## 1. Introduction

In the last few years, the outcome of patients with hematologic tumors has improved following the introduction of new agents directed against specific receptors exposed on the surface of the neoplastic cells or able to boost the immune capabilities against them, such as the Chimeric Antigen Receptor Modified T-cell (CAR-T) [1, 2]; consequently, an increasing number of these patients are now admitted to the Intensive Care Unit (ICU) both for the underlying disease and/or for its complications, including sepsis and septic shock (SS) [3, 4]. According to the 3rd International Consensus Definitions, this latter is considered a condition characterized by a multiple organ dysfunction (MODS) severe enough to increase the risk of death [5]; notably, this definition does not consider the following items: first of all the time course, the fulminant forms rapidly leading to MODS and death are lumped together with lesser aggressive ones than the different timeframes of SS, which can be characterized by an initial hyper-inflammatory condition switching later on to a reduced immune response, consisting of low-grade inflammatory state, hypercatabolism and the occurrence of secondary infections caused by multiple drug resistant germs and/or the reactivation of latent virus, such as Epstein–Barr virus, Cytomegalovirus (CMV) and Herpes virus [6, 7].

The classical symptoms of SS include fever or hypothermia, tachycardia, arterial hypotension, and other signs related to the MODS making the diagnosis relatively straightforward. Nevertheless, in patients with hematologic cancers causes other that SS can account for this clinical phenotype, including a life-threatening hyper-inflammation associated either to the underlying disease and/or to its treatment. The recognition and treatment of these conditions appear particularly challenging for the intensivist since (a) they are relatively uncommon; (b) they can occur in the very same circumstances of SS and the related symptoms could overlap; (c) the time course can be so fast that the clinical presentation could be represented by an overt MODS just from the onset; and, perhaps most important, (d) the mainstay of the treatment is based on an aggressive immunosuppression, which is contraindicated in SS.

Hereby we describe and review a case of a patient treated for a hematologic cancer in whom the admission in ICU with the diagnosis of SS appear to have been too simplistic, because other noninfectious factors may have contributed to the MODS and to the outcome.

## 2. Case Description

A 53-year-old man was admitted to the Emergency Department due to 6-hour-lasting acute abdominal pain; the first CT scan demonstrated an edematous pancreatitis and a small amount of ascites; a second CT confirmed the results at 6 h from the first one. A couple of hours after hospitalization, he was transferred to our Intensive Care Unit (ICU) because he became confused, hypotensive, and anuric. The history revealed an advanced stage non-Hodgkin lymphoma (DLCBL, stage III) discovered two months before the current admission and treated with 5 cycles of Rituximab, cyclophosphamide, doxorubicin, vincristine, and prednisone (R-CHOP), the last completed the day before the admission in ICU. On a scheduled outpatient visit occurred two weeks before this hospitalization, the patient was prescribed a 10-day course of oral valganciclovir due a reactivation of CMV, but it was suspended after 7 days due to a rise of hepatic liver enzymes and the disappearance of the viral DNA. In the ICU, the patient was intubated and mechanically ventilated; lab investigations demonstrated a deep metabolic acidosis, hyperlactatemia in association with severe leukopenia, and reduced platelet count; these abnormalities worsened in the following hours (Table 1). With the clinical suspicion of SS, IV vancomycin, meropemen, and valganciclovir were initiated, along with the administration of IgM- and IgA-enriched intravenous immunoglobulins (Pentaglobin®, Biotest, Dreieich, Germany); at the same time, a renal replacement treatment was started in association with the extracorporeal removal of sepsis mediators (Cytosorb®, Aferetica, Mirandola, Italy). The arterial hypotension was unresponsive to the administration of incremental doses of fluids, norepinephrine (peaking at 2 mcg/kg/min) together with terlipressin (100 mg) and mg 200 of hydrocortisone; a continuous administration of NaHCO_3 _failed to correct the metabolic acidosis. Despite this extremely aggressive approach, the clinical conditions further deteriorated, and the patient died six hours after the ICU admission.

**Figure 1:**
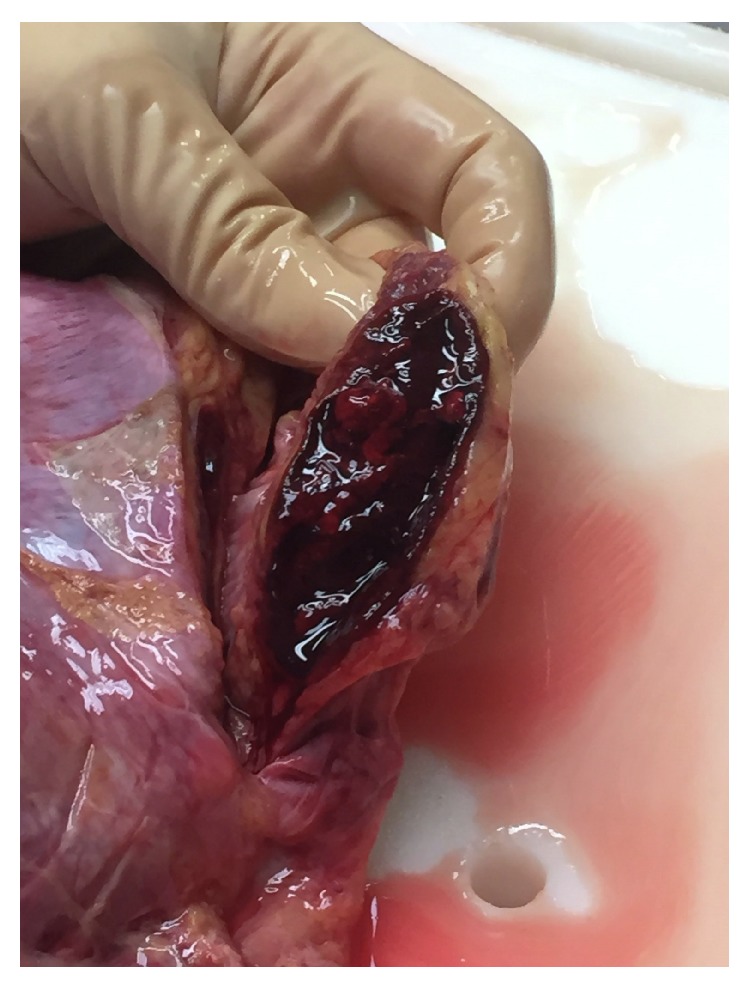
Adrenal hemorrhage.

At the autopsy, the liver and the spleen were enlarged and a massive purulent peritonitis was present in the absence of visceral perforations despite a severe mucosal ischemia that involved the enteric tract completely in the absence of mesenteric vascular obstructions; the pancreatic head was mildly edematous; the adrenal glands appeared hemorrhagic (Figure 1); the heart was dilated and presented a biventricular a diffuse subendocardial ischemia despite normal coronary arteries.

**Figure 2:**
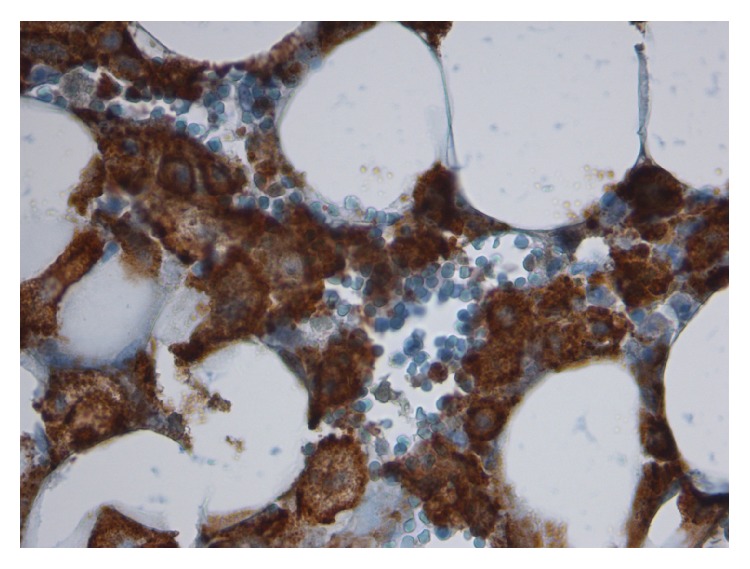
Bone marrow with extensive hemophagocytosis (CD 163, ×40).

Microscopically, the bone marrow presented an extensive hemophagocytic lymphohistiocytosis (HLH) in the absence of residual traces of the NHL (Figure 2); an *E. coli* strain sensible to the administered antibiotics was isolated from the blood culture.

## 3. Discussion

The current Surviving Sepsis Campaign (SSC) guidelines for the treatment of SS recommend the prompt administration of wide-spectrum antibiotics possibly associated with antiviral and antifungal agents in selected cases [8]. However, the diagnosis and the treatment cannot rely entirely on the microbiological findings since (a) in a high rate of cases, cultures remain sterile throughout the clinical course [9, 10]; and (b) a recent study demonstrated that in >25% of patients initially diagnosed as having SS the symptoms were caused by noninfectious diseases [11]; consequently, causes other than infections must be considered especially in the absence of any improvement despite an aggressive approach. This particularly applies to patients with hematologic tumors, in whom different factors, including the underlying disease, the related complications, and the ever-increasing use of agents interacting with the immune system can imitate SS, thus preventing the correct diagnosis and approach.

In our patient, the fulminant course of MODS could have been determined by different factors acting alone or in combination.

First, a low cardiac output (CO) could have initially occurred, determining the widespread necrosis of the enteric mucosal and the subsequent escape of bacteria and their derivates from the gut lumen, leading both the activation of septic mediators and the peritonitis. The isolation of *E. coli* in the bloodstream and the severe peritonitis in the absence of perforation supports this hypothesis, even if we could not perform an echocardiography due to the fulminating course of the MODS. The cause of the reduction of the CO is hard to identify, but it could be assumed that it was due to the anti-NHL treatment: actually, doxorubicin and the otheranthracyclines can cause a dose–dependent cardiotoxicity eventually leading to a progressive irreversible heart failure,although cases of sudden-onset cardiogenic shock have also been described [12–14]. In our patient, the cumulative dose of doxorubicin exceeded 210 mg/m^2^corresponding to an odd ratio of cardiotoxicity <7% [12]. The diffuse subendocardial ischemia observed at the post-mortem examination could be ascribed to the elevated doses of vasopressors used.

Second, although an infective trigger for the MODS is highly plausible, the further rapid deterioration of the clinical conditions despite the administration of an appropriate antibiotic treatment makes the presence of other concomitant conditions likely: actually, Vincent et al. demonstrated that in SS patients the death is associated more with a lack of improvement than with an unrelenting worsening of the clinical conditions [15]. Conversely, in our patient the MODS rapidly deteriorated since its onset and did not respond to the standard treatment of SS making the co-existence of other factors plausible.

Third, the patient underwent several cycles of a regimen that included Rituximab, a monoclonal anti-CD20 antibody largely used in the treatment of hematologic cancers and other disorders caused by chronic inflammatory conditions and an ever-increasing list of immunologic disorders [16]. The administration of rituximab has been associated with a number of early- and late-onset complications; the former include anaphylactic or allergic reactions occurring during the infusion or in the following 24 h and the latter are related to its long-term effects, including immunosuppression, renal dysfunction, and *de novo *hematologic tumors. Considering the interval from the last administration of rituximab, we guess that the profound and unresponsive hemodynamic collapse observed in our patient could be related more to the decrease of immune capabilities determined by the repeated cycles of R-CHOP than to a rituximab-induced anaphylactic reaction.

Fourth, the clinical picture could be ascribed to a hemophagocytic lymphohistiocytosis (HLH). This disturbance of the immune function exists in a familiar and in an acquired form and it is characterized by the massive production and release of inflammatory mediators caused by the dysregulated activation of T-lymphocytes, natural killer (NK)-cells, and macrophages. The familiar form is caused by the mutation of specific genes regulating the cytotoxic granule pathway leading to the impairment of the cytotoxic activity of NK cells and cytotoxic T-lymphocytes while leaving intact the production of inflammatory cytokines, whereas in the latter the underlying mechanism is less understood and has been associated with a number of factors, including rheumatologic and autoimmune disorders, viral infections, and tumors [17]; altogether, these disorders are denominated Macrophage Activation Syndrome (MAS) and the related symptoms mime SS in its hyperinflammatory phase [18]. In both forms the stimulated cells are unable to kill the invading microorganisms, yet can activate macrophages, thus triggering a process ultimately leading to an hyperinflammatory status resembling SS due to the release of elevated amounts of IFN-*γ*, TNF, lL-6, IL-10 etc., [19–21]. The acquired form of HLH is increasingly recognized in SS patients undergoing bone marrow examination even if its role in influencing the outcome is still uncertain [20, 21]. As far as the underlying disorders in critically ill adult ICU patients are concerned, in a recent study HLH occurred as a complication of hematologic malignancies (57%, mainly NHL), infections (25%, mainly CMV), or a combination of both (4%) [22]. In patients with SS, it has been hypothesized that a persisting release of inflammatory mediators in response to an uncontrolled infectious trigger can prompt the occurrence of HLH that, in turn, further increase their production thus initiating a vicious cycle ultimately leading to a MOF [23]. The diagnosis of acquired HLH is based on the fulfillment of at least of 8 criteria originally established by the Histiocyte Society for the diagnosis of the congenital form (HLH 2004) (Table 2) [22]. To overcome the limitations of using this approach in the acquired HLH, a specific score (HScore) has been developed by analyzing with a logistic regression a data set of variables drawn from ICU adult patients with confirmed HLH [23] (Table 3). In our patient, despite blood levels of ferritin being relatively low (1750 mcg/ml) as compared with those reported in other studies with values exceeding 10.000 mcg/ml [20–24], both the HLH 2004 and the HScore were high enough (5 criteria and 227, respectively, corresponding to a probability of HLH ≥95%) to make the diagnosis very likely [23]. Independently from the causes, the treatment of acquired HLH consists of the administration, alone or in combination, of immunodepressant including steroids, etoposide, or cyclosporine. Due to the fulminant time course and the diagnosis of suspected SS, we did not initiate such treatment but a blood purification technique was started as a rescue treatment to remove the sepsis mediators [25]. Recently, a number of biologic agents targeting different substances involved both in different forms of MAS have been investigated, but results from clinical trials performed in acquired HLH are lacking [26–28].

Fifth, a similar stormy clinical course is described in patients with a catastrophic antiphospholipid syndrome (CAPS), which is determined by the widespread occlusion of the microvascular network by microthrombi triggered by the Lupus anticoagulant (LAC) and the anti-cardiolipin (aCL) and anti-*β*2-glycoprotein I (a*β*2GBPI) antibodies [30]. Although the pathogenesis of CAPS is not fully clear, a number of circumstances have been associated with its occurrence, including infections, systemic lupus erythematosus, and tumors; among these latter, hematological malignancies appear particularly involved [31].The diagnosis is challenging and requires a high index of suspicion, the exclusion of other causes and the fulfillment of a number of clinical, laboratory, and pathologic criteria (Table 4). The acute treatment of CAPS consists of (1) general supportive measures (2) a rapid onset anticoagulation with iv heparin (c) a multi-faceted strategy aiming to block the production of LAC and APS or to hasten their elimination, including the administration of steroids, rituximab, plasma exchange, and immunosuppressant agents. In our patient, the absence of microvascular plugging makes the diagnosis unlikely.

Lastly, it is difficult to establish a cause-effect relationship between the adrenal hemorrhage (AH) and the MODS. Its occurrence is uncommon and is facilitated by physiological and anatomical factors including the elevated adrenal blood flow passing directly from the arteriolar network into the capillary plexus [32]. As a reassessment of the whole clinical course confirmed the absence of adrenal abnormalities at the admission CT scan, it likely occurred as a consequence of a reduced CO and SS and accounted for the failed response to vasopressors and fluid resuscitation [32, 33].

In conclusion, it is difficult if not impossible to individuate a single shooting gun responsible for the rapidly fatal MODS observed in our patient almost each potential diagnosis has its own pro- and against- factors (Table 5) as well as different and sometimes mutually exclusive approaches (Table 6); rather, it is likely that the peritonitis-induced and the SS and HLH cooperated in determining a CRS and that their combined effect led to the AH. Although the clinical presentation was suggestive also for CAPS, the biochemical and pathological findings do not confirm this diagnosis.

## 4. Conclusions

In patients with hematologic tumors, different factors can determine a MOF, including SS and/or other conditions associated either to the underlying disease and/or to its treatment; actually, it is hypothesizable that the prevalence of these latter will increase with a more widespread use of agents influencing the immune system such as of CAR-T. In our patient, the fulminant course can be likely ascribed to the purulent peritonitis likely determined by the massive bacterial translocation from the ischemic intestinal mucosa, although also other concomitant causes such as HLH could have triggered a massive release of inflammatory mediators. It could be advisable to measure the blood values of some related markers such as ferritin, LAC, aCL, and a*β*2GBPI when the hemodynamic conditions fail to respond to a sepsis-oriented treatment in order to restrict the immunosuppressive treatment to patients with a defined diagnosis.

## Figures and Tables

**Table 1:** Time course of some blood biological variables.

Variable	Timing			R.F.
	ED (6 h before ICU admission)	ICU admission	+4 h	
pH	n.a.	6.97	7.06	7.38–7.42
pCO_2_(mmHg)	n.a.	68.4	53.1	35–45
PaO_2 _/ FIO_2 _(mmHg)	n.a.	94	66	≥400
HCO_3_^−^ (mEq/L)	n.a.	16.4	14.8	23–25
Lactate (mEq/L)	n.a.	6.7	10.8	1–2
Hb (g/dl)	14.1	12.5	13.5	13–16
WBC/ml	1820	650	350	4000–11000
Platelets/ml	131.000	89.000	14.000	180–350
IRN	n.a.	1.53	1.84	0.78–1.2
aPTTr	n.a.	0.95	2.44	0.76–1.18
Fibrinogen (mg/dl)	n.a.	272	219	180–400
D-dimer (mcg/LFEU)	n.a.	2.11	16000	<5
Glycemia (mg/dl)	132	80	72	90–110
Creatinine (mg/dl)	1.40	2.76	2.90	0.8–1.1
BUN (mg/dl)	66	80	83	15–50
ALT(U/L)	29	n.a.	65	0–40
AST (U/L)	55	n.a.	69	0–40
Amilase (U/L)	35	79	80	8–53
Ferritin mcg/m	n.a.	1750	n.a.	14–300
Triglycerydes (mg/dl)	n.a.	180	n.a.	<170
C-reactive protein (mg/dl)	36.2	142	n.a.	<5.0
Total bilirubin (mg/dl)	n.a.	0.69	0.79	0.70–1.20

ED: emergency department; R.F.: reference values; n.a.: not available; FEU: fibrinogen equivalent unit.

**Table 2:** Diagnostic criteria of HLH.

(i) Molecular diagnosis consistent with HLH	Case
(ii) Or 5 of the following criteria		
(1) Fever	+
(2) Splenomegaly	+
(3) Cytopenia affecting ≥ 2 lineages	+
(a) Hemoglobin <9 g/dl	−
(b) Platelets <90.000/ml	+
(c) Neutrophils <1000/ml	+
(4) Hypertrigliceridemia and/or hypofibrinogemia	−
(a) Triglycerides >265 mg/dl	−
(b) Fibrinogen <150 mg/dl	−
(5) Hemophagocytosis in bone marrow, spleen or nodes (∗)	+
(6) Low/absent NK cell activity	n.a.
(7) Ferritin ≥500 mcg/ml	+
(8) sCD25 (sIL2R) ≥2400 U/ml	n.a.

n.a: not available.

**Table 3:** HScore.

Variable	Criteria for scoring	Case
Immunosuppression	0: no	18
	18: yes		

Temperature (°C)	0 (<38.4)	33
	33 (38.4–39.4)		
	49 (>39.4)		

Organomegaly	0 (no)	38
	23 (Hepatomegaly or splenomegaly)		
	38 (Hepatomegaly + splenomegaly)		

n. of cytopenias	0 (1 lineage)	24
	24 (2 lineages)		
	34 (3 lineages)		

Ferritin (ng/ml)	0 (<2000)	0
	35 (2000–6000)		
	50 (>6000)		

Triglyceride (mmol/L)	0 (<1.5)	30
	30 (1.5–4)		
	64 (>4)		

Fibrinogen (g/L)	0 (>2.5)	30
	30 (≤2.5)		

Serum ALT	0 (<30)	19
	19 (≥30)		

Hemophagocytosis in B.M.	0 (no)	35
	35 (yes)		

Total score	**337**	**227**

B.M.: bone marrow.

**Table 4:** Diagnostic criteria for CAPS (from [29]).

Preliminary criteria	Case
(1) Evidence of involvement of ≥3 organs, system and/or tissues	+
(2) Development of clinical manifestation simultaneously or in <1 week	+
(3) Evidence of small vessel occlusion in at least 1 organ or tissue	+
(4) Lab evidences of LAC and/or aCL and a*β*2GBPI	n.a.

*Definite CAPS*	
(i) All 4 criteria	−

*Probable CAPS*	
(i) All 4 criteria, 2 organs, system and/or tissues involved	−
(ii) All 4 criteria, except for the lack of lab evidence due to the early death of patients never tested for APS	−
(iii) Criteria 1, 2, and 4	−
(iv) Criteria 1, 3, and 4 and development of a third event between one week and one month after the initial presentation despite anticoagulation	n.a.

n.a: not available.

**Table 5:** Possible differential diagnoses of fulminant MODS.

Diagnosis	Pro	Against	Likelihood
Septic shock	(1) Immusuppression	(1) Fulminant time course of MODS	H
	(2) Blood culture positive for *E. coli*	(2) Not responding to standard anti-SS treatment		

Rituximab-induced cytokine release syndrome	(1) Clinical picture resembling SIRS	(1) Interval >24 h between treatment and onset of symptoms	L

Acquired hemophagocytic lymphohistiocytosis	(1) NHL	none	H
	(2) Recent CMV infection			
	(3) 5/8 preliminary diagnostic criteria fulfilled			
	(4) Elevated HScore			

Catastrophic anti phospholipid syndrome	(1) Recent NHL	(1) Diagnostic criteria unfulfilled	L

Acute adrenal failure	(1) Failed response to volume resuscitation and vasopressors	(1) No hyponatremia	H
	(2) Autopsy findings			

H: high; L: low; I: intermediate.

**Table 6:** Possible therapeutic strategies.

Condition	Therapy
Septic shock	(1) Antibiotics
	(2) Drainage of septic foci
	(3) Cardiorespiratory and renal support
	(4) Steroids (debated)
	(5) Blood purification techniques

CRS (whatever cause)	(1) Treatment of the cause
	(2) Immunosuppression
	(3) Blood purification techniques

Acquired hemophagocytic lymphohistiocytosis	(1) Immunosuppression
	(2) Etoposide
	(3) Extracorporeal Blood purification

Catastrophic anti phospholipid syndrome	(1) Immunosuppression
	(2) IvIg
	(3) Anticoagulation
	(4) Plasmaexchange
